# The next 10 years of behavioural genomic research

**DOI:** 10.1002/jcv2.12112

**Published:** 2022-11-04

**Authors:** Robert Plomin

**Affiliations:** ^1^ King's College London Institute of Psychiatry Psychology and Neuroscience London UK

**Keywords:** causal modelling, genetics, genomics, nosology, polygenic scores

## Abstract

**Background:**

The explosion caused by the fusion of quantitative genetics and molecular genetics will transform behavioural genetic research in child and adolescent psychology and psychiatry.

**Methods:**

Although the fallout has not yet settled, the goal of this paper is to predict the next 10 years of research in what could be called *behavioural genomics*.

**Results:**

I focus on three research directions: the genetic architecture of psychopathology, causal modelling of gene‐environment interplay, and the use of DNA as an early warning system.

**Conclusion:**

Eventually, whole‐genome sequencing will be available for all newborns, which means that behavioural genomics could potentially be applied ubiquitously in research and clinical practice.

## Graphical abstract

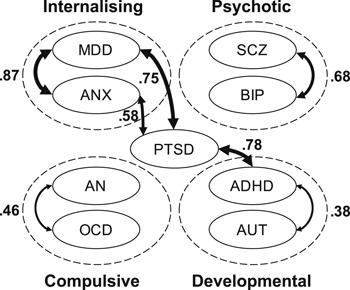

Three transformative developments for behavioural genomic research in the next 10 years are described. First, behavioural genomic research will use dimensional measures to reveal the hierarchical genetic architecture of psychopathology. A second direction for behavioural genomic research will be to investigate causa models of GE interplay. Third, polygenic scores will be used as an early warning system in childhood to predict profiles of adult psychopathology, which will eventually transform clinical practice.

**Key points**

- During the next 10 years, the fusion of quantitative genetics and molecular genetics will transform behavioural genetic research.
- Behavioural genomics will reveal the genetic architecture of psychopathology.
- Causal modelling of GE interplay will be a focus of behavioural genomic research.
- Polygenic scores will be used in childhood to predict profiles of adult psychopathology.

To appreciate the next 10 years of behavioural genetic research, it is useful to begin by looking back at the past decade when the two worlds of genetics, quantitative genetics and molecular genetics, fused in a synthesis that could be labelled *behavioural genomics*. Although the two worlds of genetics were both born more than a century ago, they went their separate ways for most of the century. Following on from Francis Galton (1865), quantitative genetics focused on complex traits such as behavioural traits that were presumed to be influenced by many genes of small effect (Fisher, 1918). A century of quantitative genetic research showed that all behavioural traits, including psychiatric disorders, are substantially heritable, with an average heritability of 50% (Polderman et al., 2015). In contrast, understanding Gregor Mendel's mechanisms of heredity (Mendel, 1866) was the goal of molecular genetics, which focused on single‐gene disorders. The goal of molecular genetics was to identify the chromosomal location of these genes and to understand how they worked.

The two fields converged as advances in molecular genetics made it possible to go beyond single‐gene effects to investigate complex traits influenced by many genes. The origins of this development began in the 1970s with the ability to sequence DNA's nucleotide bases. By the turn of the 21^st^ century, these techniques identified the sequence of most of the three billion nucleotide base pairs in the human genome. This led eventually to the discovery of millions of inherited differences in DNA sequence. In the 1990s, thousands of studies reported associations between behavioural traits and variants in ‘candidate’ genes, typically neurotransmitter genes thought to be involved in behavioural pathways. Candidate‐gene studies genotyped only a few DNA variants because genotyping was expensive and slow. However, these studies failed to replicate (e.g., Border et al., 2019; Chabris et al., 2012) – they were genomics' contribution to the replication crisis, committing every sin in the catalogue of questionable research practices (Ritchie, 2021).

In 1996, the idea emerged that association studies could be made systematic if thousands of DNA variants across the genome were genotyped (Risch & Merikangas, 1996 ). However, genome‐wide association (GWA) seemed a dream because few DNA variants had been identified and genotyping was expensive and slow. In 2003, the Human Genome Project published the sequence of most of the 3 billion steps in the spiral staircase of DNA and soon discovered millions of variants in DNA sequence. The problem of the expense of genotyping each DNA variant was solved in the mid‐2000s by the DNA microarray, which can genotype hundreds of thousands of DNA variants for an individual quickly, accurately, and inexpensively. These arrays are called *SNP chips* because they genotype the most common type of DNA variant, a single nucleotide polymorphism (SNP), and because they are analogous to silicon chips in computers. In the next 10 years, SNP chips will be replaced by whole‐genome sequencing in which the sequence of the four‐letter alphabet of DNA will be assessed for the three billion nucleotide base pairs in the genome, thus uncovering all inherited DNA differences between individuals, not just SNPs.

The SNP chip paved the way for GWA analyses. In 2007, the first major GWA analysis included 2000 cases for each of seven major disorders and compared SNP allele frequencies for these cases versus controls (The Wellcome Trust Case Control Consortium, 2007). Replicable associations were found but they were few in number and small in effect size. Hundreds of GWA reports appeared over the next decade with similar results across the behavioural and biological sciences (Visscher et al., 2017), including childhood behaviour problems such as ADHD (Demontis et al., 2019), autism (Grove et al., 2019), and anorexia nervosa (Watson et al., 2019). These findings led to the realisation that the biggest effect sizes were much smaller than anyone anticipated – risk ratios were less than 1.1 for case‐control studies and the variance explained for dimensional traits was less than 0.1%. This meant that complex traits were extremely polygenic and that thousands of SNPs would need to be identified to account for heritability. It also meant that huge sample sizes would be needed to detect these miniscule effects.

Genome‐wide association and the ensuing recognition that the heritability of behavioural traits is caused by extreme polygenicity brought the two worlds of genetics together during the past decade, creating a synthesis that could be designated *behavioural genomics*. Energy unleashed from this fusion will fuel research in child and adolescent psychology and psychiatry during the next 10 years and beyond. Most of this energy comes from two new categories of tools that will play a major role in research in the next 10 years.

First, instead of using the specialised samples of twins and adoptees to estimate the heritability of traits and genetic correlations between them, behavioural genomics provides two different methods to estimate heritability and genetic correlation. The first method, called Genome‐wide Complex Trait Analysis (GCTA or GREML), uses SNP chip genotyping data for samples of at least several thousand unrelated individuals (Yang et al., 2011). For each pair of individuals, GCTA compares the pair's overall SNP similarity to their similarity on a trait. Although each pair of unrelated individuals' SNP similarity can only vary from only −2.5% to +2.5% (which excludes pairs who are even fifth‐degree relatives), across samples of thousands of individuals, this provides millions of pair‐by‐pair comparisons, which produces a powerful estimate of heritability. This estimate is called SNP heritability because it is limited to heritability estimated by the SNPs on the SNP chip. Genetic correlations are estimated by comparing each pair's SNP similarity to their cross‐trait similarity.

SNP heritabilities are about 25% for psychopathology, which is about half the heritability estimates from twin studies (Cross‐Disorder Group of the Psychiatric Genomics Consortium, 2013b). This ‘missing heritability’ occurs because SNP heritability is limited to the common SNPs genotyped on current SNP chips, which also creates a ceiling for discovery in GWA research. Most SNPs are not common, and rare SNPs appear to be responsible for much of the missing heritability, at least for height (Wainschtein et al., 2022).

The second method, called LD score regression, estimates heritability and genetic correlations just using summary statistics from GWA studies rather than requiring SNP chip data for each individual as in GTCA (Bulik‐Sullivan et al., 2015). The essence of the method is the regression of association effect sizes of SNPs on their distance apart on a chromosome (linkage disequilibrium) because SNPs closer together on a chromosome should show similar effect sizes for true associations. LD score regression yields SNP heritability estimates similar to GCTA.

GCTA and LD score regression both document missing heritability. However, when it comes to genetic correlations, GCTA and LD score regression yield results similar to twin estimates. In other words, there is no ‘missing genetic covariance’, for reasons explained elsewhere (Trzaskowski et al., 2013). LD score regression has been combined with structural equation modelling from twin analyses to model the genetic structure of genetic correlations among multiple traits, a method called Genomic Structural Equation Modelling (Genomic SEM) (Grotzinger et al., 2019).

The second category of tools is polygenic scores. Although the heritability of complex traits is due to many SNPs of small effect, it is possible to add up these small effects weighted by each SNP's effect size from GWA summary statistics to create polygenic scores (Allegrini et al., 2022; Wray et al., 2021). Polygenic scores for any GWA target trait can be created in any sample of unrelated individuals for whom GWA genotype data are available. It should be noted that, although huge samples are needed for genome‐wide association studies, a polygenic score that predicts 10% of the variance only needs a sample size of 60 to detect its effect with 80% power (*p* = 0.05, one‐tailed). Polygenic scores are the topic of the third section of this paper.

Because the fallout has not yet settled from the fusion of the two worlds of genetics, it is difficult to predict research directions during the next 10 years. What is clear is that this will be an exciting time for behavioural genomic research (Larsson, 2021a). I chose three substantive issues that I predict will stand out as major areas of research during the next 10 years: the genetic architecture of psychopathology, causal modelling of gene‐environment interplay, and the use of DNA as an early warning system. My aim was to make provocative rather than pedestrian predictions, and ones that are sufficiently specific to have their validity evaluated in 10 years' time.

Although the pace of advances in behavioural genomics is exhilarating, it should be noted that most of the major discoveries so far in behavioural genetics have come from quantitative genetic studies using twin and adoption designs that originated a century ago (Plomin et al., 2016). Quantitative genetic research will continue to make important contributions because it estimates total genetic influence and is intrinsically as much about the environment as it is about genetics. It is heartening to see that results from behavioural genomics generally confirm those from quantitative genetics.

## THE GENETIC ARCHITECTURE OF PSYCHOPATHOLOGY

In the next 10 years, behavioural genomic research will move beyond documenting the inadequacies of current nosology to reveal the genetic architecture of psychopathology in childhood, adolescence, and adulthood.

Psychiatry has been having a crisis of confidence about its classification of disorders, which is based on symptoms (Zachar & Kendler, 2017). Attempts to re‐classify disorders on the basis of presumed causes such as neural processes have produced a welter of findings about biomarkers but no breakthroughs in terms of re‐classification (Pacheco et al., 2022). Genetics is different because, unlike other biomarkers whose correlations with behavioural traits cannot be interpreted causally, genetics has a unique causal status in that there can be no backward causation. That is, events in the environment, behaviour or the brain cannot change inherited DNA differences (Plomin & von Stumm, 2022).

Genetics is not everything – it accounts for about half of the variance for psychopathological traits and all other behavioural traits (Polderman et al., 2015) – but it is almost everything systematic about psychopathology (Plomin, 2018). For psychopathology, the nongenetic half of the variance is not due to systematic effects of family environment shared by children growing up in the same family. The salient environmental influences are nonshared and seem to be unsystematic, perhaps idiosyncratic and stochastic (Gidziela, Malanchini, et al., 2022).

For these reasons, it makes sense scientifically to focus on the genetic architecture of psychopathology, even though causes are not necessarily related to cures and the impact on treatment remains to be seen. Although phenotypic structures reflect genetic structures for cognitive abilities and personality, domains whose structures were built psychometrically from the ground up, the genetic architecture of psychopathology so far looks very different from current symptom‐based diagnoses, whose origins are more historical than empirical.

In this section, I will provide a brief overview of how genetic and genomic research has revealed the inadequacies of current nosology, despite a circularity in that this research has necessarily relied on current diagnoses. I will then consider how research in the next 10 years can reveal the genetic architecture of psychopathology.

## GENETIC CORRELATIONS BETWEEN DISORDERS

For decades, genetic research has revealed dramatic examples of the inadequacy of current nosology. One of the oldest examples from twin studies is that anxiety and depression show genetic correlations near 1.0, indicating that these two disorders do not differ genetically (Middeldorp et al., 2005). Another example is that the first GWA studies of psychopathology, which focused on the psychotic disorders of schizophrenia and bipolar disorder, found that many of the same SNPs were associated with both disorders (The International Schizophrenia Consortium, 2009). This finding was shocking because schizophrenia and bipolar disorder are among the oldest and most distinctive diagnoses and, at that time, were differentiated in the first tier of DSM‐4 diagnosis so that people could not receive both diagnoses.

**FIGURE 1:**
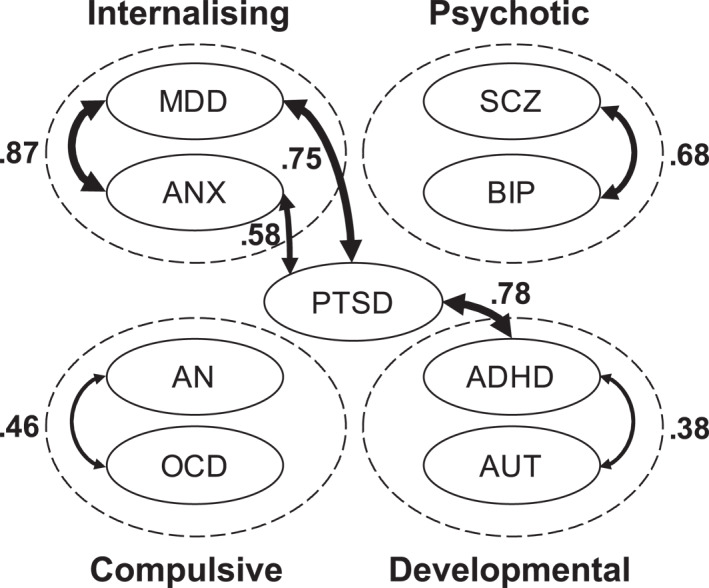
Patterns of genetic correlations for nine psychiatric disorders derived from GWA summary statistics (LD score regression). Derived from results reported by Grotzinger et al. (2022). To simplify the presentation, two disorders in the Grotzinger et al. analysis – problematic alcohol use and Tourette syndrome – are not depicted here because they are largely independent of the other disorders.

A great advantage of behavioural genomic analysis is that LD score regression (Bulik‐Sullivan et al., 2015) can estimate genetic correlations between disorders from GWA summary statistics without the need to assess the disorders in the same individuals or to have access to genotyping data for individuals. I will focus on the most recent report of this type (Grotzinger et al., 2022), which replicates and extends previous research (e.g., Bulik‐Sullivan et al., 2015; Cross‐Disorder Group of the Psychiatric Genomics Consortium, 2013a; Lee et al., 2019; Selzam et al., 2018), including twin studies (Martin et al., 2018). Genetic correlations were estimated from LD score regression using case‐control GWA summary statistics for 11 psychiatric disorders with the largest GWA samples (See Figure 1.).

The highest genetic correlation, 0.87, was found between major depressive disorder (MDD) and anxiety disorders (ANX). Also confirming previous research, the genetic correlation between schizophrenia (SCZ) and bipolar disorder (BIP) was 0.68. For the childhood disorders of attention‐deficit/hyperactivity disorder (ADHD) and autism (AUT), the genetic correlation was 0.38. Another cluster involves anorexia nervosa (AN) and obsessive‐compulsive disorder (OCD), which correlate genetically 0.46. A surprising finding was that post‐traumatic stress disorder (PTSD) correlated strongly with MDD (0.75), ANX (0.58), and ADHD (0.78). In summary, in line with previous research, there is a great deal of genetic overlap between disorders.

Genomic SEM was used to factor analyse these genetic correlations. These analyses identified four broad factors that correspond to the clusters of genetic correlations indicated by the patterns of genetic correlations illustrated in Figure 1: internalising (MDD and ANX), psychotic (SCZ and BIP), developmental (ADHD and AUT), and compulsive (AN and OCD). PTSD loaded on both the internalising and developmental factors.

A second important behavioural genomic discovery is that, even beyond these four genetic clusters, there is a positive manifold among all 11 disorders. Of the 55 pairwise genetic correlations, all but six were statistically significant and the average genetic correlation for the 55 correlations was 0.28. For example, ADHD and AUT correlated genetically with SCZ (0.20, 0.25, respectively), BIP (0.19, 0.13), MDD (0.47, 0.40), and ANX (0.36, 0.35), in addition to genetic correlations of 0.78 and 0.47 with PTSD.

This genetic overlap among disorders reflects a transdiagnostic factor that has been called *p* (Caspi & Moffitt, 2018), analogous to *g*, the general factor of cognitive ability. Phenotypic research during the past decade shows substantial comorbidity among disorders in adulthood (Caspi et al., 2014; Lahey et al., 2012; Wright et al., 2013) and childhood (Allegrini, Cheesman, et al., 2020; Sallis et al., 2019). Genetic and genomic studies have shown that this ubiquitous comorbidity is mostly genetic in origin (McLaughlin et al., 2012; Pettersson et al., 2016; Selzam et al., 2018).

Behavioural genomic analyses provide the strongest evidence for a genetic p factor among psychiatric disorders (Waszczuk et al., 2020) but not for neurological disorders (Anttila et al., 2018). The Grotzinger et al. (2022) Genomic SEM analysis tested the fit of a hierarchical model of p, analogous to the widely accepted hierarchical model of g. In the hierarchical model, the latent variable of p extracts genetic covariance among the four latent factors illustrated in Figure 1. The hierarchical model fit the data well, and the p factor explained 30–55% of the genetic variance in the four factors. The p factor has a broad reach, yielding substantial genetic correlations with diverse behavioural traits including personality (agreeableness, −0.73; neuroticism, 0.78; subjective wellbeing, −0.75), lifetime symptoms (depression, 0.94; mania, 0.87, psychosis, 0.90, stress‐related disorders, 0.91), suicide attempts (0.67), family relationship satisfaction (−0.58), deprivation (0.54), insomnia (0.42) and cannabis use (0.43).

Thus, despite analysing genetic correlations for putatively distinct disorders diagnosed according to current nosology, genetic overlap is the rule rather than the exception. In other words, currently nosology does not reflect the genetic architecture of psychopathology.

### Disorders are dimensions

A second finding with far‐reaching implications for the genetic architecture of psychopathology is that common disorders are dimensions. That is, there are no genetically distinct disorders, just continuous dimensions (Plomin et al., 2009). A dimensional approach does not denigrate the clinical and societal problems at the extremes of these dimensions nor the clinical necessity of identifying these extremes. The point is that there is nothing to be gained scientifically by reifying the extremes of normal dimensions as aetiologically distinct categories.

Much phenotypic evidence supports this view that disorders are dimensions (Krueger et al., 2018). Genetic support comes from twin studies which report that diagnosed disorders yield substantial genetic correlations with corresponding quantitative traits (Geschwind & Flint, 2015; Martin et al., 2018; Taylor et al., 2019).

**FIGURE 2:**
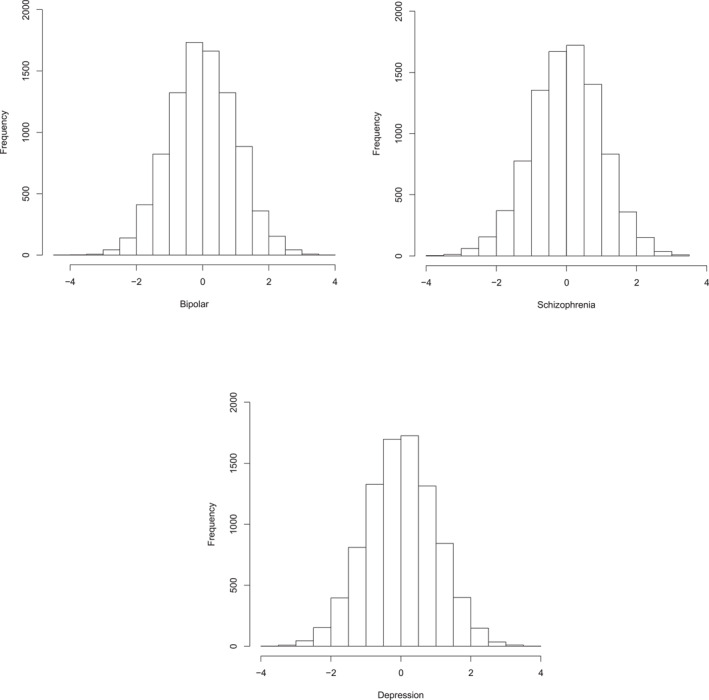
Distributions of polygenic scores for schizophrenia, bipolar disorder and depression for 10,346 individuals in the Twins Early Development Study (Rimfeld et al., 2019). Polygenic scores were standardised with a mean of 0 and a standard deviation of 1 because of the varying ranges of the polygenic scores. The polygenic scores were created for individuals in TEDS using summary statistics from the following case‐control GWA meta‐analyses: schizophrenia (Pardiñas et al., 2018), bipolar disorder (Mullins et al., 2021), and depression (Howard et al., 2019).

Behavioural genomic research provides definitive evidence in two ways. The first is so obvious that it is easy to overlook its importance: Polygenic scores derived from case‐control GWA studies of diagnosed disorders are perfectly normally distributed, showing no indication of a breakpoint or threshold for disorder (See Figure 2.). Although these normal distributions necessarily follow from the central limit theorem of probability, as polygenic scores are used to predict genetic risk for psychopathology, it will be impossible to ignore the fact that this risk is continuous.

More specific support for the view that disorders are dimensions comes from behavioural genomic research that shows substantial genetic correlations between diagnosed disorders and quantitative traits (Martin et al., 2018). For example, the genetic correlation between ADHD diagnosis and quantitative traits, for which comparable measures exist for diagnosis and traits, exceeds 0.90 (Demontis et al., 2019). A polygenic score derived from a case‐control GWA analysis of ADHD also predicts ADHD symptoms in a community sample (Taylor et al., 2019). Major depressive disorder yields genetic correlations greater than 0.90 with quantitative measures of depressive symptoms (Anttila et al., 2018; Wray et al., 2018). Comparisons for schizophrenia and bipolar disorder are more difficult because comparable trait measures are less straightforward (Martin et al., 2018).

Categorical models have been less dominant in child psychopathology than in adulthood (Caspi & Moffitt, 2018), due in part to the high frequency of behaviour problems in childhood and the availability of dimensional measures of behaviour problems. This suggests that research in childhood could be in the forefront of research revealing the genetic architecture of psychopathology.

### Revealing the genetic architecture of psychopathology

Genetic correlations between diagnoses and evidence that common disorders are dimensions clearly demonstrate that current diagnoses do not reflect the genetic architecture of psychopathology. In the next 10 years, behavioural genomic research will move beyond documenting the inadequacies of current nosology to reveal the genetic architecture of psychopathology.

The major roadblock is that most GWA studies have used case‐control designs that are based on traditional diagnostic nosology, which makes it difficult to go beyond this nosology because these studies are limited to the specific diagnosis for which cases were selected. In the near term, GWA studies will consider symptoms of diagnoses within case‐control studies. However, this is limited by the same circularity – for example, if a diagnosis requires three symptoms, it is not possible to use such cases to assess the genetic associations between the three symptoms.

The sound way to reveal the genetic architecture of behavioural problems is to build it from the ground up, using batteries of behavioural problems assessed as dimensions in samples of unrelated individuals. A starting point is the phenotypic structure of psychopathology. An important example is the Hierarchical Taxonomy of Psychopathology (HiTOP) project (Kotov et al., 2017), whose goal is to group symptoms phenotypically using dimensional measures. Emerging from this research is a hierarchical model with a p factor on top of broad factors of externalising, internalising and psychotic experiences. The extent to which the phenotypic structure of symptoms coincides with the genetic structure is an open, but empirical, issue (Lahey et al., 2021; Waszczuk et al., 2020). In the meantime, it would be useful to sharpen up genomic studies of specific disorders by correcting for p using a technique called GWAS‐by‐subtraction (Demange et al., 2021).

Instead of focussing on traits viewed as symptoms of current diagnoses of behaviour problems, a more radical approach is to consider problems as the extremes of normal dimensions of personality (Widiger, 2011), such as the extremes of activity, attention, impulsivity, shyness, fearfulness, and anger. One advantage of a personality perspective is that, unlike psychopathology, the architecture of personality has been a focus of psychometric research from the beginning, leading to a model of adult personality dominated by the ‘Big 5’ factors of OCEAN (openness to experience, conscientiousness, extraversion, agreeableness, and neuroticism). A general p‐like factor has also been proposed (van der Linden et al., 2010). In childhood, it would be worth revisiting temperament theories from decades ago, which focus on early appearing personality traits (Goldsmith et al., 1987; Shiner et al., 2012). For example, activity level is not well represented in measures of adult personality, but it is impossible to ignore activity level in childhood (Buss & Plomin, 1984).

Another advantage of a personality perspective is that personality traits, unlike measures of symptoms, are normally distributed, as are polygenic scores. These normal distributions will draw attention to both ends of the distribution. Measures of symptoms presuppose that one end of the distribution is the problem – for example, too much activity and insufficient attention. However, for most personality distributions, the other extreme can also be problematic, but these are ignored because they are not as disruptive – too little activity or too much attention. The normal distribution of polygenic scores will foster research on the both the low and high ends of the distribution.

During the next 10 years, I predict that the outlines of the genetic architecture of psychopathology in childhood, adolescence, and adulthood will be revealed by behavioural genomic research, unconstrained by current nosology. Research to date suggests that the structure at each age is hierarchical with p on top, and that it will be built from dimensional measures, with psychopathology defined as the quantitative extremes of these dimensions.

Although the structure of psychopathology is likely to differ in childhood, adolescence and adulthood, another important research direction is to capitalise on the fact that inherited DNA sequence variants do not change during development so that it is possible to identify traits in childhood that best predict adult psychopathology (Akingbuwa et al., 2020; Allegrini, Cheesman, et al., 2020; Gidziela, Rimfeld, et al., 2022; Riglin et al., 2022). In other words, this suggests that, in addition to outlining the genetic structure of psychopathology in childhood, the genetic structure of psychopathology in adulthood could be used in childhood to explore what could be called the predictive structure of childhood psychopathology. For example, p in adulthood, which will be different from p in childhood, could be used to probe the predictive structure of p in childhood. The third section of this paper considers this direction for research in greater detail.

The clinical utility of knowing the genetic structure of psychopathology remains to be seen, although exciting advances are looming such as transdiagnostic treatments (Caspi & Moffitt, 2018) and quantitative approaches to treatment rather than ‘cures’. Turning the question of clinical utility around, what is to be gained clinically by pretending that current disorders are aetiologically distinct and that they are dichotomous rather than dimensional? Regardless of its clinical utility, the scientific value of an aetiologically accurate architecture seems beyond doubt.

## CAUSAL MODELLING OF GE INTERPLAY

In the next 10 years, a major direction for behavioural genomic research will be causal modelling of GE interplay.

A major advance in quantitative genetics was to go beyond estimating genetic and environmental influence to investigate their interplay, which includes interactions (GxE) and correlations (rGE) between genetic and environmental effects. Traditional quantitative genetic methods, such as twin and adoption studies, are only able to provide indirect glimpses of GE interplay (Plomin et al., 1977). An overview of three subsequent stages in research on GE interplay is instructive because it shows why GE interplay will be a focus of behavioural genomic research during the next 10 years.

The first stage incorporated measures of the environment in quantitative genetic designs. For GxE, twin studies found examples in which heritability of psychopathology differed as a function of the environment, a very limited form of GxE (Dick, 2011), which might be called heritability × E interaction to highlight its limitation. Adoption studies offered wider scope for GxE studies because the genotype of adopted children can be inferred from their biological parents and the environment can be assessed in the adoptive home. Examples have been reported in which the effect of this genetic index on childhood psychopathology was moderated by characteristics of the adoptive home (Knopik et al., 2017).

The main finding from this first stage of research on GE interplay was about the importance of rGE rather than GxE: Measures of the environment widely used in psychology show significant and substantial genetic influence, about 25% on average (Kendler & Baker, 2007), called the *nature of nurture* (Plomin & Bergeman, 1991). Furthermore, correlations between environmental measures and children's outcomes were shown to be mediated genetically, about 50% on average (Avinun & Knafo, 2014; Plomin, 1994). These findings documented the importance of rGE. Moreover, twin and adoption studies can distinguish passive rGE in which parents pass on family environments correlated with their genotypes, evocative rGE in which parents respond to the children's genetic propensities, and active rGE in which children modify or create environments correlated with their genetic propensities (Plomin, 1994). In general, evidence for passive rGE was found for cognitive traits. Causal modelling of rGE was enabled by designs that include parents of twins and children of twin parents (McAdams et al., 2014) and by longitudinal data in which time is used to leverage causality (Berry & Willoughby, 2017).

The second stage of research on GE interplay incorporated measured genotypes in candidate genes in addition to measured environments. Hundreds of reports of GxE appeared but most failed to replicate (Dick et al., 2015). Some correlations between candidate genes and environmental measures also indicated rGE, but these too had a poor track record for replication (Jaffee & Price, 2007). The fundamental problem with candidate‐gene research is lack of power: we now know that single DNA variants hardly ever account for as much as 0.1% of the variance in the population. Candidate‐gene studies were vastly underpowered to detect such effect sizes, and as a result most reported associations were false positives (Border et al., 2019; Chabris et al., 2012; Duncan & Keller, 2011).

The third stage of research on GE interplay is behavioural genomics, which exploits GWA genotyping data for hundreds of thousands of SNPs on SNP chips from large samples. Much of this research attempts to confirm the existence of GxE and rGE using strategies similar to the candidate‐gene era but substituting polygenic scores for candidate genes (Plomin & Viding, 2022). It will be important to avoid repeating the questionable research practices that led to failures to replicate in the candidate‐gene era. Systematic multivariate studies will help (Allegrini, Karhunen, et al., 2020), and replication will be key.

The focus of this section is on methods that have enabled new possibilities for causal modelling of GE interplay (Pingault et al., 2018). The overall goal of causal modelling in relation to GE interplay is to disentangle ‘direct’ effects of genes and environments from rGE.

### Mendelian randomisation

Mendelian randomisation is a method for causal modelling that uses genetics to identify causal effects of modifiable environmental factors on outcomes rather than investigating rGE itself (Davey Smith & Ebrahim, 2003). The strongest proof of causality is an experiment with random assignment to conditions, as in randomised controlled trials, but this is often precluded by ethical issues. Rather than randomising participants into different levels of treatment, Mendelian randomisation capitalises on the fact that individuals are randomised by genotypes because genotypes are randomly allocated from parents to offspring. If genotypes are strongly associated with an environmental factor (exposure) as well as with an outcome, the genetic ‘instrument’ can be used as a proxy for randomly assigned exposure as it affects the outcome. A path model can isolate the causal effect of the exposure if the model meets demanding assumptions: the genetic instrument is correlated with the outcome exclusively through its effect on the exposure and is not correlated with confounding factors that influence the correlation between the exposure and the outcome.

Mendelian randomisation was initially applied to single‐gene effects and dichotomous ‘exposures’ and outcomes, which are conditions not relevant to the complexity of psychopathology, but it has been extended to polygenic scores and quantitative environmental factors and outcomes (Pingault et al., 2018). However, the assumptions of Mendelian randomisation are much more daunting with polygenic scores which are embedded in a complex web of rGE (Koellinger & de Vlaming, 2019; Krapohl et al., 2017). Although the method has been reported to identify some modifiable risks, for example, for depression (Choi et al., 2020), as yet no strong causal paths have been discovered for psychopathology (Richmond & Davey Smith, 2022).

Mendelian randomisation analyses will be added to many behavioural genomic studies of GE interplay during the next 10 years. However, I predict that few modifiable environmental risks will be found and replicated for psychopathology because of the complex web of rGE.

### Back to families

Although the ability of GWA to identify genetic effects in large samples of unrelated individuals is its strength, returning to family data enriches causal modelling of GE interplay. GWA analyses based on unrelated individuals and the polygenic scores derived from them include rGE effects as well as between‐family effects such as ethnicity, socioeconomic status (SES) and assortative mating. Finding that polygenic scores correlate with traits within families moves a step closer to causal genetic effects because it controls for all between‐family effects. That is, if the sibling with the higher polygenic score has a higher trait score than the other sibling, the association between the polygenic score and the trait cannot be due to between‐family effects. Finding within‐family correlations eliminates passive rGE effects because both siblings passively receive their genes and environments from their parents. Within‐sibship analyses also eliminate all other between‐family effects such as SES and assortative mating because siblings also share these effects. Although within‐sibship effects have been called ‘direct effects’, they only control for passive rGE, not evocative or active rGE.

To the extent that polygenic score correlations for unrelated individuals exceed within‐family polygenic score correlations this indicates the effect of between‐family factors. So far, it appears that between‐family effects contribute to polygenic score prediction for cognitive traits, which are impacted by SES and assortative mating, but not for behaviour problems (Selzam et al., 2019). A novel approach directly assesses within‐sibship effects by conducting GWA analyses of sibling differences rather than individual differences among unrelated individuals (Howe et al., 2022).

Adding parental GWA genotypes to create trios consisting of two parents and a child can further tease apart rGE. The first research in this area showed that parental polygenic scores predict their children's traits independent of the children's polygenic scores (Bates et al., 2018; Kong et al., 2018). These effects have been labelled ‘indirect effects’ or ‘genetic nurture’, but again only passive rGE is controlled in these analyses. Such indirect effects have so far only been found for cognitive traits, not behaviour problems (Willoughby et al., 2021) and it has been suggested that these effects of ‘genetic nurture’ are actually between‐family stratification effects (Nivard et al., 2022).

These analyses do not specify which environmental factors are responsible, but measured environments can be incorporated in this approach. For example, ADHD polygenic scores of mothers and their children both correlate with household chaos, indicating rGE contributions from both mothers and children (Agnew‐Blais et al., 2022). However, children's ADHD polygenic scores continue to correlate with household chaos after controlling for mothers' ADHD polygenic scores. This controls for passive rGE effects and suggests that children contribute to household chaos by evocative or active rGE. Research like this incorporating environmental measures is needed to go beyond passive rGE to investigate the more general processes of evocative and active rGE, although the major hurdle here is that current measures of the environment are not well suited to assess evocative rGE and especially active rGE.

Causal modelling of GE interplay using polygenic scores is limited by their effect size in predicting behaviour. In contrast, GCTA (Yang et al., 2011) estimates heritability and genetic correlations by comparing overall SNP differences from GWA genotyping with phenotypic differences pair by pair for thousands of individuals. GCTA can be used in these ways to investigate GE interplay (Choi et al., 2022). Combined with trios, GCTA can also separate direct and indirect genetic effects on behaviour problems (Eilertsen et al., 2022).

Given the torrent of new methods and opportunities to apply them, causal inference about GE interplay is certain to be a focal area of behavioural genomic research during the next 10 years.

## USING POLYGENIC SCORES AS AN EARLY WARNING SYSTEM

In the next 10 years, polygenic scores for psychopathology will predict more than 10% of the variance and will be used in childhood to predict profiles of adult vulnerabilities.

As indicated in the previous section, extracting causality from essentially correlational data is difficult, despite the advances in behavioural genomic methods to tease apart GE interplay in causal models (Pingault et al., 2022). However, polygenic scores can be used to predict behavioural traits without knowing anything about intervening causal processes. The goal of prediction is to account for as much variance as possible without regard for explanation (Larsson, 2021a). It has been argued that, from the perspective of prediction, it is not even necessary to disentangle the extent to which the prediction is due to rGE, assortative mating, or population stratification (Plomin & von Stumm, 2022).

Polygenic scores have a unique causal status because, as noted earlier, correlations between polygenic scores and behaviour are not subject to backward causation. This means that polygenic scores that predict adult psychopathology can be used in infancy as an early warning system to predict psychopathology in adulthood. In other words, polygenic score predictions are the same for DNA from an infant and from an adult. Although behaviour problems in childhood and adolescence are important in their own right, predicting adult psychopathology in childhood will be a focus for research in the next 10 years because of its clinical implications for intervention and prevention (Larsson, 2021b).

**FIGURE 3:**
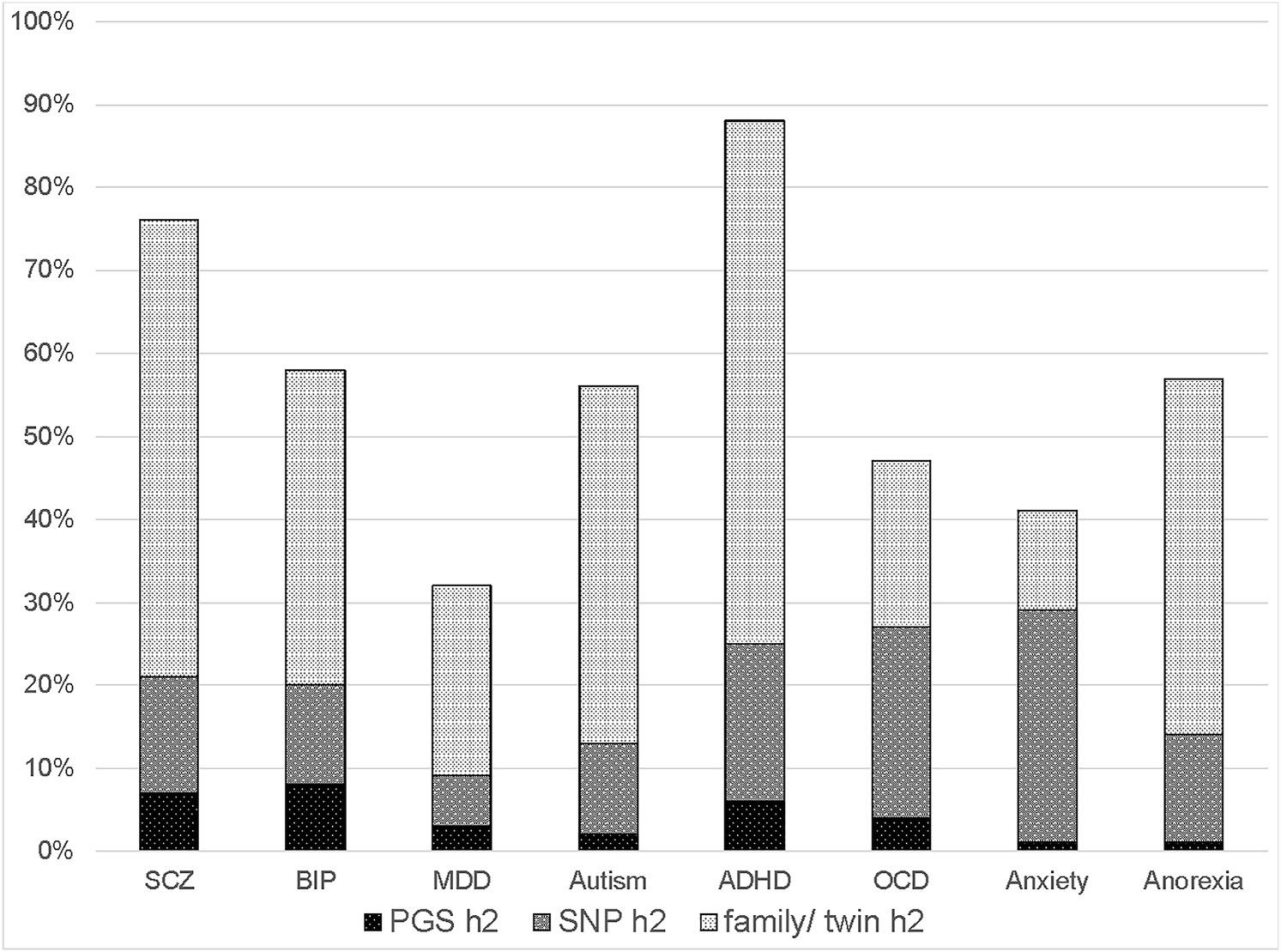
Liability heritability estimates for eight psychiatric disorders based on polygenic scores (PGS h^2^), SNP chip GWA genotypes (SNP h^2^), and family/twin h^2^. PGS h^2^ is the liability variance predicted by the polygenic score (usually Nagelkerke’s *R* ^2^). SNP h^2^ is the liability variance estimated from GWA summary statistics using LD score regression. Family h^2^ is the liability model‐fitting estimate from family or twin data. PGS h^2^ references: schizophrenia (SCZ; Trubetskoy et al., 2022); bipolar disorder (BIP; Stahl et al., 2019); major depressive disorder (MDD; Howard et al., 2019); autism (Grove et al., 2019); attention deficit/hyperactivity disorder (ADHD; Demontis et al., 2019); obsessive‐compulsive disorder (OCD; Heinzel et al., 2021); anxiety (Purves et al., 2020); anorexia (Watson et al., 2019). SNP h^2^ references: (Grotzinger et al., 2022). Family h^2^ references: SCZ, BIP, MDD (Song et al., 2015); autism (Colvert et al., 2015); ADHD (Larsson et al., 2014); OCD (Mataix‐Cols et al., 2013); anxiety (Song et al., 2015); anorexia (Bulik et al., 2010).

Figure 3 shows polygenic score heritability estimates for eight psychiatric disorders with the largest GWA meta‐analyses. The average polygenic score heritability estimate is 4%, ranging from 1% for anxiety and anorexia to 8% for bipolar depression. However, these are logistic regressions that discriminate cases and controls to estimate heritability of liability, which is a hypothetical construct that assumes a normal distribution of risk underlying the diagnosis and does not translate directly to variance explained in the population.

### The 10% target

During the next 10 years, I predict that polygenic scores will explain more than 10% of the variance of psychopathology. I hope by that time psychopathology will be assessed dimensionally so that the target of predicting 10% of the variance in the population will be clearly in view rather than obfuscated by liability statistics based on case‐control data.

The ability to predict 10% of the variance is a watershed in three ways. First, predicting 10% of the variance is off the scale of most predictions in the behavioural sciences. However, this is often overlooked because of the lingering preoccupation with statistical significance rather than subjecting findings to the harsh spotlight of effect size.

Second, polygenic scores with such effect sizes predict large differences at the extremes. Using the most recent polygenic score for schizophrenia that predicts 7% of the liability as an example (Trubetskoy et al., 2022), individuals in the highest centile are 39 times more likely to be diagnosed as schizophrenic as compared to individuals in the lowest centile of the polygenic score. The top centile is 5.6 times more likely to be diagnosed when compared to the remaining 99% of individuals.

Third, effect sizes of this magnitude are large enough to be ‘perceptible to the naked eye of a reasonably sensitive observer’ (Cohen, 1988, p. 26). Nonetheless, 10% of the variance is equivalent to a correlation of 0.32, an oval‐shaped scatterplot that reflects the limitations of the probabilistic nature of prediction when correlations are less than 1.0.

### Reaching the 10% target

Predicting 10% of the variance is a conservative target because polygenic scores in the cognitive realm already predict more than 10% of the variance for the quantitative traits of intelligence (Plomin & von Stumm, 2018), educational attainment (Okbay et al., 2022), and educational achievement (Allegrini et al., 2019).

Figure 3 indicates that there is plenty of headroom for increasing the predictive power of polygenic scores for psychopathology. The ultimate ceiling for polygenic score prediction is twin heritability, but the current ceiling is SNP heritability because GWA results and the polygenic scores derived from them rely on the common SNPs assessed on extant SNP chips. On average for these eight disorders, polygenic score heritability is only one‐fifth of the SNP heritability. This missing heritability gap can be narrowed through the brute force of larger GWA case‐control studies, but a bigger pay‐off is likely to come from GWA research using dimensional measures that correspond to the genetic architecture of psychopathology. Support for the hierarchical dimensional approach comes from a recent GWA study of a broad factor of externalising behaviours using Genomic SEM that yielded a polygenic score that predicted 10% of the variance (Karlsson Linnér et al., 2021). Another analytic strategy is to use longitudinal assessments that can capture the dynamic nature of psychopathology and capitalise on the fact that genetics is largely responsible for age‐to‐age continuity (Gidziela, Rimfeld, et al., 2022).

The second type of missing heritability is the gap between SNP heritability and estimates of heritability using family and twin designs. Figure 3 shows that, on average, SNP heritability is only 37% of family and twin estimates of heritability. Narrowing this missing heritability gap can substantially raise the ceiling for polygenic score prediction. This will require different technologies such as whole‐genome sequencing, which can increase SNP heritability by adding rarer DNA variants to the common variants assessed on current SNP chips (Wainschtein et al., 2022).

### Implications and applications

Polygenic scores are already transforming research in developmental psychology. They will democratise genomics by making it possible for all researchers to add genomics to their programme of research, which will produce novel findings that permeate research and clinical practice. No longer are special samples of twins or adoptees needed for genomic analysis, just DNA. Behavioural assessment is not even needed because polygenic scores can be used as genetic proxies. For example, the genetics of cognitive abilities can be brought to bear without costly assessment using polygenic scores for cognitive ability. Finally, polygenic scores can be employed as genetic predictors in any moderately sized sample of unrelated individuals. As noted earlier, a polygenic score that predicts 10% of the variance only needs a sample size of 60 to detect its effect with 80% power. These are the reasons why all major longitudinal cohort studies have obtained DNA.

The most exciting application of polygenic scores will be to transform clinical work from symptoms to causes, from treatment to prediction and prevention, from one‐size‐fits‐all interventions to individually tailored interventions based on treatment response, and, as highlighted earlier, from qualitative diagnoses to quantitative dimensions (Plomin, 2018). For these reasons, during the next 10 years, as polygenic scores become more predictive, they will begin to make a difference clinically as they are used in childhood to predict profiles of vulnerabilities for adult psychopathology.

‘I am almost certain that complete genome sequencing will become part of newborn screening in the next few years…. It is likely that within a few decades people will look back on our current circumstance with a sense of disbelief that we screened for so few conditions’ (Collins, 2010, p. 50). This prediction was made by Francis Collins, who directed the Human Genome Project and then served as director of the US National Institutes of Health until 2021. There are signs that his prediction is belatedly becoming true. For example, it has been reported that by 2025 China expects to conduct whole‐genome sequencing on half of its 10 million babies born each year (Metzl, 2019). It is not a question of whether infants are genotyped at birth – for decades, newborns in most countries have been screened for a few single‐gene mutations. The cost of this screening is comparable to genotyping on a SNP chip, but rather than switching to SNP chips, whole‐genome sequencing seems inevitable as the cost continues to drop, perhaps to £100 (Pennisi, 2022), because whole‐genome sequencing captures all DNA variation. Newborn screening focuses on single‐gene mutations for medical disorders but polygenic scores will surely be incorporated as they become more predictive of problems later in life. Also inevitable is the use of polygenic scores in prenatal screening, at least for couples undergoing in vitro fertilisation (von Stumm & Plomin, 2021).

Although difficult ethical complications of newborn genotyping need to be addressed, a practical implication for developmental researchers is that genotype data could potentially be available for all children, without the need to obtain DNA or genotype it. That is, once children are genotyped on a SNP chip or by whole‐genome sequencing, their genotypes could be used to create any polygenic score. Thus, developmental researchers and clinicians in the future may be able to add polygenic scores to their research at no cost other than analytic costs of creating polygenic scores, which are becoming routinised.

Another direction for research during the next 10 years is to create more predictive polygenic scores by moving beyond Eurocentric samples to more diverse samples (Peterson et al., 2019). This effort has begun, for example, with the US project *All of Us,* which was launched in 2018 and plans to enrol more than one million people of diverse ancestry (https://allofus.nih.gov/about), and the UK project, *Our Future Health*, which plans whole‐genome sequencing for 5 million individuals of diverse ancestry (https://ourfuturehealth.org.uk/).

## CONCLUSION

The outpouring of opportunities created by behavioural genomics is unparallelled in the behavioural sciences, as illustrated by the three transformative developments for research in the next 10 years outlined here. Understanding the genetic architecture of psychopathology, investigating genetic and environmental causal paths, and using polygenic scores as an early warning system will also eventually transform clinical practice.

## GLOSSARY

**Allele frequency** Population frequency of a DNA variant.

**Candidate genes** A gene whose function suggests that it might be associated with a trait.

**DNA** Deoxyribonucleic acid, the double‐stranded molecule that encodes genetic information. The two strands are held together by hydrogen bonds between two of the four nucleotide bases, with adenine bonded to thymine and cytosine bonded to guanine.

**DNA microarray** Miniature slides with hundreds of thousands of short single‐stranded DNA sequences that serve as probes to detect SNPs. Commonly called SNP chip.

**Genetic correlation** A statistic indexing the extent to which genetic influences on one trait are correlated with genetic influences on another trait independent of the heritabilities of the traits.

**Genome‐wide association (GWA)** An association study that assesses DNA variants throughout the genome, typically for SNPs assessed on DNA microarrays.

**Genome‐wide complex trait analysis (GCTA)** Estimates SNP heritability and genetic correlations explained by all SNPs for a trait rather than testing the association between any particular SNP and the trait.

**Genomic structural equation modelling (Genomic SEM)** A statistical framework for modelling multivariate genetic variance structures.

**Genotyping** Assessing an individual's pair of alleles at a particular locus.

**Heritability** The proportion of phenotypic variance among individuals that can be attributed to inherited DNA differences in a particular population.

**Human genome** All three billion DNA nucleotide base pairs in our species, packaged in 23 pairs of chromosomes.

**Linkage disequilibrium (LD) score regression** Estimating SNP heritability and genetic correlations from GWA summary statistics by regressing association effect sizes of SNPs on their distance apart on a chromosome (linkage disequilibrium).

**Missing heritability** The difference between variance explained by polygenic scores (PGS heritability) and family estimates of heritability. Another type of missing heritability is the difference between PGS heritability and SNP heritability, which creates a ceiling for PGS heritability.

**Molecular genetics** The investigation of the effects of specific genes at the level of DNA.

**Nature of nurture** Genetic influence on measures of the environment and on the covariance between environmental measures and behavioural traits.

**Nonshared environment** Environmental influences that do not contribute to resemblance between family members.

**Nucleotide base** A single step in the spiral staircase of the DNA double helix consisting of hydrogen bonds between two of the four nucleotide bases, with adenine bonding to thymine and cytosine bonding to guanine. The DNA code is a sequence of three nucleotide base pairs that codes for one of the 20 amino acids, which are the building blocks of proteins.

**Polygenic** A trait influenced by many genes.

**Polygenic score** A genetic index of a trait for an individual that is the sum across the genome of thousands of the individual's increasing alleles associated with the trait, usually weighted by the effect size of each SNP's association with the trait, based on GWA summary statistics for the trait.

**Quantitative genetics** A theory of multiple‐gene influences that, together with environmental variation, result in in quantitative (continuous) distributions of traits. Quantitative genetic methods such as twin and adoption methods estimate genetic and environmental contributions to variance of traits and covariance between traits in a population.

**Shared environment** Environmental influences that make family members similar.

**Single‐gene disorder** Caused by variants in a single gene, which yields Mendelian patterns of inheritance.

**Single‐nucleotide polymorphism (SNP)** The most common type of DNA variant which involves a difference in a single nucleotide.

**SNP heritability** Heritability estimated directly from DNA differences (SNPs) between individuals.

**Whole‐genome sequencing** Determining the complete sequence of nucleotide base pairs for a genome.

## AUTHOR CONTRIBUTION

**Robert Plomin**: Conceptualization, Writing – original draft.

## CONFLICTS OF INTEREST

The author has declared that he has no competing or potential conflicts of interest.

## ETHICAL CONSIDERATIONS

Not applicable to this article.

## Data Availability

Data sharing not applicable to this article as no datasets were generated or analysed during the current study.
